# Audit of Child and Adolescent Admissions to an Adult Psychiatric Unit: Addressing Service Gaps and Legal Compliance

**DOI:** 10.1192/bjo.2025.10624

**Published:** 2025-06-20

**Authors:** Praveen Kumar, Kevin Edassery, Parth Dhirawani, Soma Tadi

**Affiliations:** 1 City Hospital, Aberdeen, United Kingdom; 2 Royal Cornhill Hospital, Aberdeen, United Kingdom; 3 NHS Grampian, Aberdeen, United Kingdom

## Abstract

**Aims:** Guidance from the Mental Welfare Commission for Scotland (2013) and the Scottish Government’s Best Practice Guideline for Admission to Adult Mental Health Wards for Under 18s (2013) states that young people should be admitted to age-appropriate settings, with admission to adult psychiatric units occurring only in exceptional circumstances. The United Nations Convention on the Rights of the Child (UNCRC) also advocates for age-specific mental health services.

This audit examined the frequency, legal compliance, and contributing factors of under-18 admissions to an adult psychiatric unit over six months. It aimed to assess adherence to mental health legislation, including the Mental Health (Care and Treatment) (Scotland) Act 2003, and identify areas for service improvement.

**Methods:** A retrospective review was conducted of all under-18 admissions to the adult psychiatric unit at Royal Cornhill Hospital, Aberdeen, Scotland, between June and December 2024. Data on demographics, referral sources, diagnoses, legal status, duration of stay, and completion of the ADM form (mandatory for notifying Scotland’s Mental Welfare Commission of under-18 admissions) were analysed.

**Results:** Five adolescents (aged 13–17) were admitted during the audit period. Primary diagnoses included substance misuse-induced psychosis (n=2), disordered eating (n=1), acute stress reaction (n=1), and personality disorders (n=1). Legal status at admission varied: 60% (n=3) were voluntary, while 40% (n=2) were detained under a Short-Term Detention Certificate.

Critical non-compliance with legal safeguards was identified: only 40% (n=2) had ADM forms completed, breaching statutory requirements under the Mental Health (Care and Treatment) (Scotland) Act 2003. Most referrals originated from regions lacking dedicated CAMHS beds, underscoring geographic inequities in service provision. The average stay was 20.4 days (range: 7–42), reflecting delays in transferring patients to age-appropriate units.

**Conclusion:** These findings highlight systemic failures in delivering developmentally appropriate care, compounded by non-adherence to legal protocols. Extended admissions to adult wards risk therapeutic harm and violate safeguards for vulnerable minors. Urgent measures are required to address service gaps, including investment in dedicated adolescent inpatient units, mandatory staff training on legal compliance, and improved regional coordination to streamline referrals. Policymakers must prioritise equitable access to CAMHS beds and reinforce accountability mechanisms to prevent inappropriate placements. This audit underscores the imperative for Scotland to align its psychiatric care practices with both ethical standards and mental health legislation, ensuring vulnerable young people receive safe, rights-based care.

